# PDE4 inhibitor rolipram represses hedgehog signaling *via* ubiquitin-mediated proteolysis of GLI transcription factors to regress breast cancer

**DOI:** 10.1016/j.jbc.2025.108239

**Published:** 2025-01-27

**Authors:** Arka Bagchi, Anuran Bhattacharya, Analava Bera, Deblina Basak, Urmi Chatterji, Arunima Biswas

**Affiliations:** 1 Cell and Molecular Biology Laboratory, Department of Zoology, University of Kalyani, Nadia, West Bengal, India; 2 Cancer Research Laboratory, Department of Zoology, University of Calcutta, Kolkata, West Bengal, India

**Keywords:** breast cancer, phosphodiesterase inhibitor, cAMP, PKA, Hedgehog signaling pathway, GLI

## Abstract

Aberrant activation of the Hedgehog (Hh) signaling pathway positively correlates with progression, invasion, and metastasis of several cancers, including breast cancer. Although numerous inhibitors of the Hh signaling pathway are available, several oncogenic mutations of key components of the pathway, including Smoothened, have limited their capability to be developed as putative anticancer drugs. In this study, we have modulated the Hh signaling pathway in breast cancer using a specific Food and Drug Administration–approved phosphodiesterase 4 inhibitor rolipram. The results indicated that increased levels of cAMP-dependent PKA, because of the treatment with rolipram on MCF-7 and MDA-MB-231 cells, induced PKA-mediated ubiquitination of glioma-associated oncogene homolog 2 full length (GLI2FL) and GLI3FL, leading to their transformation to respective repressor forms. This in turn reduced the level of GLI1 (glioma-associated oncogene homolog 1) transcription factor in a time-dependent manner. We have also shown that elevated levels of PKA reduced the level of phosphorylated glycogen synthase kinase 3**β**, which is known to augment PKA-mediated ubiquitination of GLI2FL and GLI3FL. Rolipram treatment also impaired wound healing and migration in both cell lines and significantly reduced tumor weight and volume in tumor-bearing mice. Histological analysis showed a reduction in multinucleated cells and cellular infiltration in the lungs of rolipram-treated mice. Moreover, rolipram decreased GLI1 levels in tumors by enhancing cAMP–PKA signaling. These findings suggest that rolipram effectively inhibits the Hh pathway downstream of Smoothened, offering potential as a therapeutic strategy for controlling breast cancer progression and metastasis, including both hormone-responsive and triple-negative subtypes.

The Hedgehog (Hh) signaling pathway is very critical during adult homeostasis following repair and injury (1) and aberrant activation of the same modulates multiple aspects of tumorigenesis (2). It is therefore not surprising that together with a major role in maintenance of the self-renewal capacity of adult somatic stem cells, the Hh signaling has been widely implicated in cancer and cancer stem cell function and maintenance. Since the Hh signaling pathway contributes to tumor growth, maintenance, and recurrence, understanding the role that Hh signaling plays in regulating breast cancer cells is of vital importance. A wide array of small molecules that target the Hh downstream component Smoothened (Smo), including the canonical Hh inhibitor cyclopamine and the Smo antagonist vismodegib, have been shown to affect tumor progression. However, oncogenic mutations of Smo have limited their use as potential Hh inhibitors (3). Identification of distal signaling mediators downstream of Smo could thereby provide new therapeutic opportunities for Hh-dependent malignancies.

The downstream signaling cascade of the canonical Hh pathway is mediated by nuclear translocation of GLI transcription factors, which consequently initiate transcription of specific target genes (4). An emerging understanding of the activation of GLI transcription factors has identified phosphorylation of GLI by PKA for both its nuclear translocation and repressor function. Several studies have already demonstrated that PKA can lead to either increase or decrease of Hh target gene expression in different tissues (5). Moreover, this phosphorylation leads to priming of adjacent phosphorylation events, which are mediated by glycogen synthase kinase 3β (GSK3β) and casein kinase (6). Therefore, the activity of GSK3β also plays a pivotal role in the processing of GLI proteins. On the other hand, PKA-mediated phosphorylation is grossly related to the intracellular level of cAMP, which is known to be associated with tumor progression and cell differentiation (7) and is widely put forward as a therapeutic target for autoimmune and inflammatory diseases (8). cAMP is regulated by adenylate cyclase, which produces cAMP from adenosine triphosphate, and phosphodiesterases (PDEs), which hydrolyzes cAMP to adenosine monophosphate (9). In humans, there are at least 11 isoforms of PDEs that are utilized as a therapeutic target against various diseases because of their tissue-specific distribution (8). Therefore, to modulate the fate and abundance of cAMP in breast tissues in a compartmentalized manner, tissue-specific distribution of PDEs may be exploited as a potential therapeutic approach.

Previous studies by our group demonstrated that PDE4, one of the several isoforms of PDEs, is overexpressed in breast tumor tissues compared with normal breast tissues. Inhibition of PDE4 by rolipram, a previously used drug for Alzheimer’s disease and a potential antidepressant (10), altered the fate of MDA-MB-231 triple-negative breast cancer (TNBC) cells and the cancer stem cell population, which contribute to the relapse of tumors *via* the cAMP–PKA axis (11). Since the cAMP–PKA axis has been shown to perturb the Hh signaling in relevant tissues during embryogenesis (12), we have attempted to elucidate the involvement of Hh signaling components in response to rolipram in both hormone-responsive and TNBC cells. In our previous work, we have also shown the efficacy of rolipram when used with conventional taxol group chemotherapeutic agents like paclitaxel against TNBC cells and breast cancer stem cells (brCSCs). This study will further elucidate the functional aspects of rolipram so as to develop it as a repurposed drug, to be used with existing chemotherapeutic agents, for synergistic effects on breast cancer cells.

## Results

### Rolipram induces cytotoxicity in hormone-responsive and TNBC breast cancer cell lines by modulating intracellular cAMP levels

**Figure 1:**
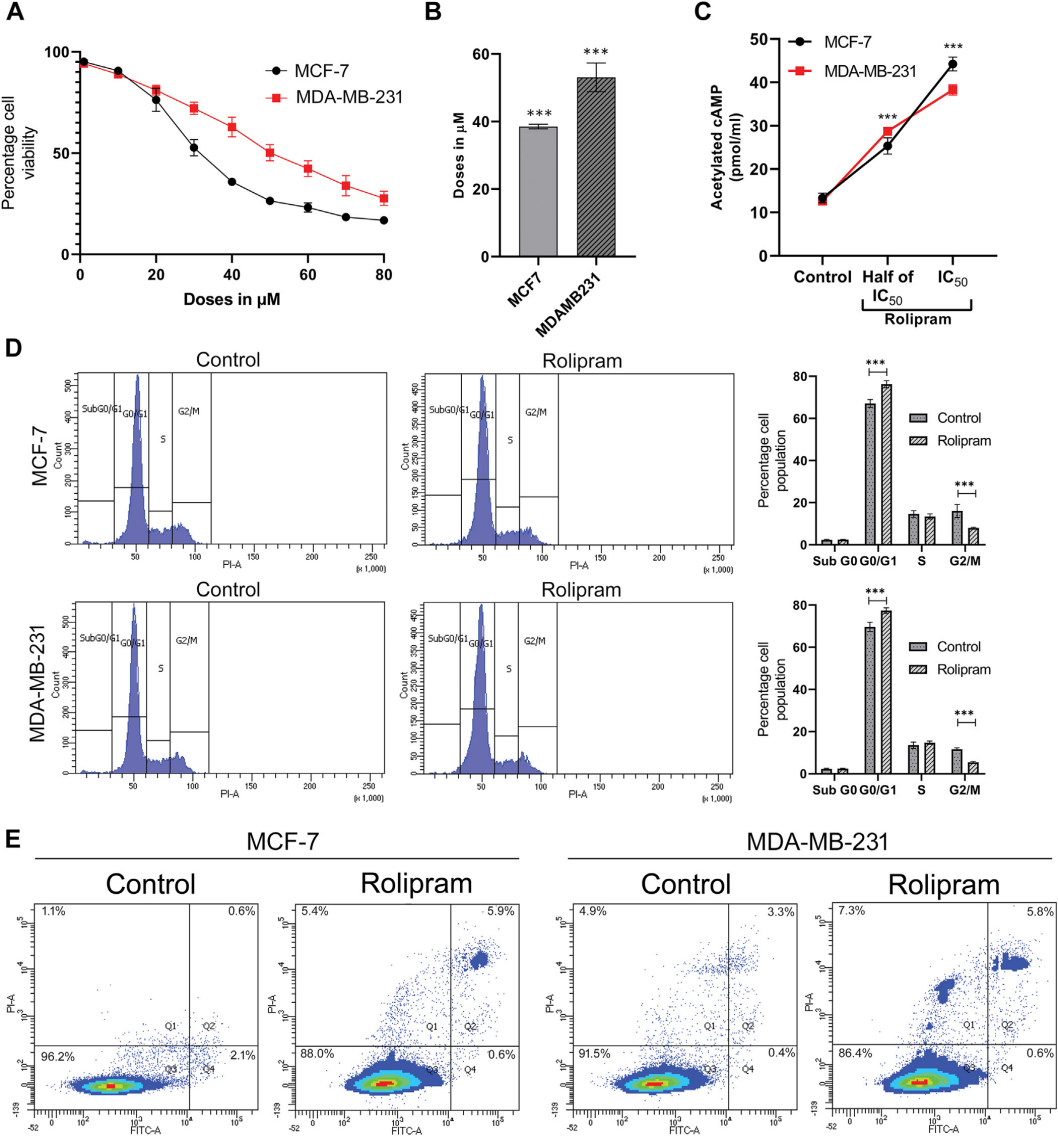
**Effect of rolipram on cell viability.***A*, dose-dependent regression in cell viability of MCF-7 and MDA-MB-231 cells following treatment with different doses of rolipram. *B*, bar representation of the IC_50_ values of rolipram in MCF-7 and MDA-MB-231 cell lines obtained from MTT assay. *C*, change in the levels of intracellular cAMP of MCF-7 and MDA-MB-231 cells after treatment with rolipram at respective IC_50_ and half IC_50_ doses for 24 h. *D*, histogram plots obtained from flow cytometric analysis of DNA content of untreated MCF-7 and MDA-MB-231 cells (control) and cells treated with rolipram at their respective IC_50_ doses for 24 h along with bar representation of the percentage of cell population at each stage of cell cycle. *E*, plots obtained from flow cytometric analysis of MCF-7 and MDA-MB-231 cells after treatment with IC_50_ doses of rolipram for 24 h, followed by double staining with Annexin V-FITC and PI. Each experiment was repeated at least three times independently (∗∗∗*p* < 0.001). MTT, 3-[4,5-dimethylthiazol-2-yl]-2,5 diphenyl tetrazolium bromide; PI, propidium iodide.

The effect of rolipram on both hormone-responsive and TNBC cell lines was assessed by treating respective cell lines with increasing doses of rolipram (1–100 μM). A dose-dependent reduction in cell survivability was observed in both MCF-7 and MDA-MB-231 cells. The concentration for 50% growth inhibition (IC_50_) was determined to be 38 μM for MCF-7 cells and 53 μM for MDA-MB-231 cells (Fig. 1, *A* and *B*, *p* < 0.001). The intracellular level of cAMP simultaneously increased in a dose-dependent manner in both the cell lines, when treated with rolipram for 24 h. Rolipram increased the cAMP level to 24.02 pmol/ml at half IC_50_ dose and 43.12 pmol/ml at IC_50_ dose in MCF-7 cells compared with 14.1 pmol/ml in untreated control. Similarly, in MDA-MB-231 cells, cAMP level increased up to 28.35 and 37.41 pmol/ml, at half IC_50_ and IC_50_ doses, respectively, compared with 12.55 pmol/ml in untreated cells (Fig. 1*C*) (*p* < 0.001). Simultaneously, analysis of the DNA content of MCF-7 and MDA-MB-231 cell lines after 24 h of treatment with rolipram at its respective IC_50_ doses revealed that rolipram significantly increased the G_0_/G_1_ cell population in both the cell lines (*p* < 0.001) (Fig. 1*D*). Moreover, double staining of MCF-7 and MDA-MB-231 cells with Annexin V-FITC and propidium iodide (PI) revealed that there is 8.1% increase in the dead cell population in MCF-7 cell line (*p* < 0.001) and 5.2% increase in MDA-MB-231 cells (Fig. 1*E*).

### Rolipram induces PKA-mediated ubiquitination of GLI2 and GLI3 to regress Hh signaling

**Figure 2:**
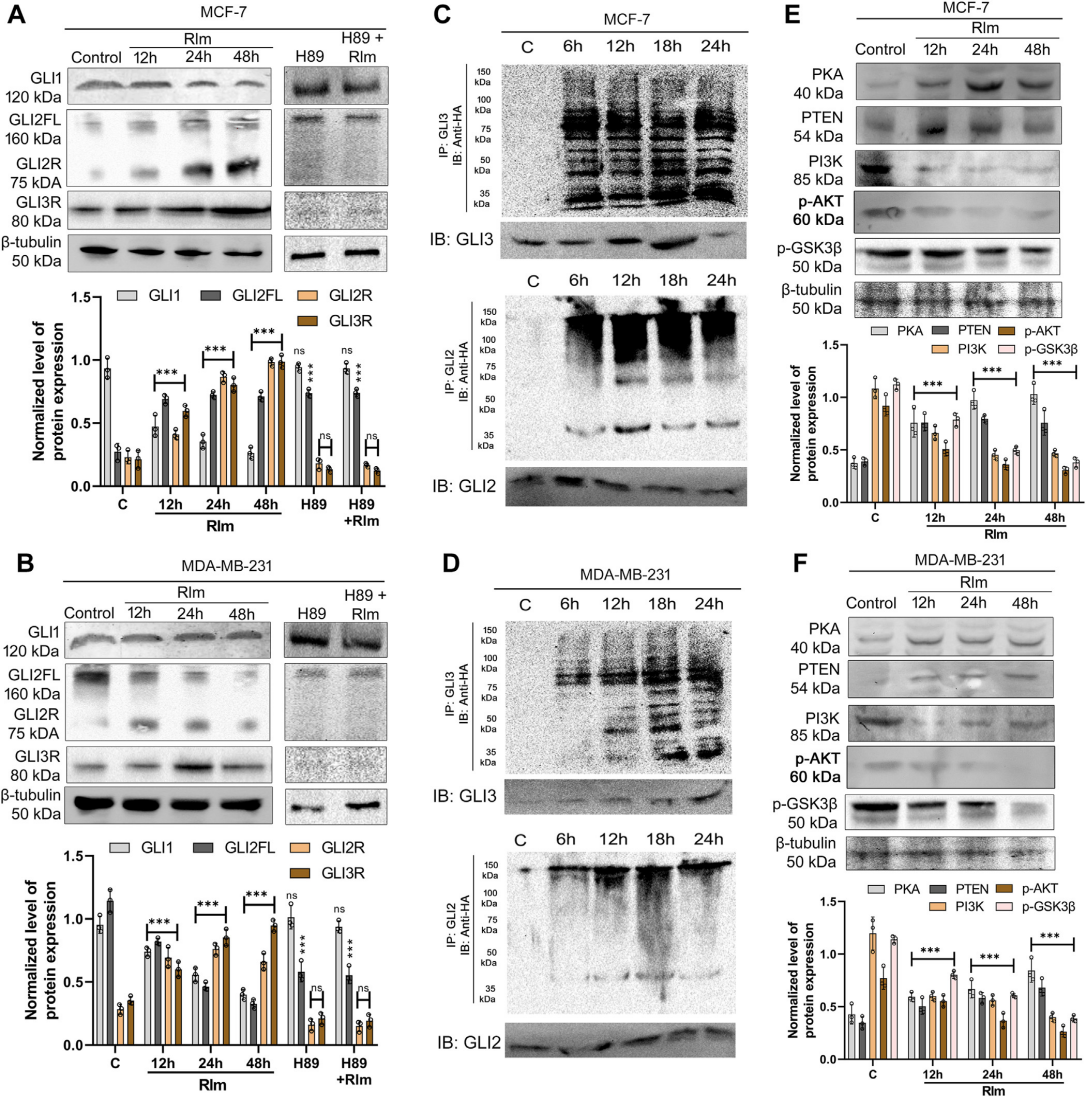
**Modulation of hedgehog signaling**. *A*, expression of GLI1, GLI2FL, GLI2R, and GLI3R at different time points after treating MCF-7 cells with rolipram at its IC_50_ dose as well as H89. The expressions of these proteins were also observed after cotreatment with H89 and rolipram at 24 h time point. *B*, expression of GLI1, GLI2FL, GLI2R, and GLI3R at different time points after treating MDA-MB-231 cells with rolipram at its IC_50_ dose, H89, and a combination of H89 and rolipram. The treatment of H89 and H89–rolipram treatment was done for 24 h. The quantification of the blots in (*A*) and (*B*) was done after normalizing the band intensity of the control sets from these figures with the respective control sets shown in Fig. S1. *C*, ubiquitination status of GLI2 and GLI3 following treatment of MCF-7 cells with IC_50_ dose of rolipram at different time points of 6, 12, 18, and 24 h. *D*, ubiquitination status of GLI2 and GLI3 following treatment of MCF-7 cells with IC_50_ dose of rolipram at 6, 12, 18, and 24 h. Expression of PKA, PTEN, PI3K, phospho-Akt, and phospho-GSK3β at different time points after treating MCF-7 cells (*E*) and MDA-MB-231 cells (*F*) with rolipram at its IC_50_ doses. All protein expressions were normalized against β-tubulin. GLI1, glioma-associated oncogene homolog 1; GLI2FL, glioma-associated oncogene homolog 2 full length; GLI2R, glioma-associated oncogene homolog 2 repressor; GLI3R, glioma-associated oncogene homolog 3 repressor; GSK3β, glycogen synthase kinase 3β; PTEN, phosphatase and TENsin homolog.

Since rolipram increased cAMP-dependent PKA, the association of GLI transcription factors with the PKA signaling and the association of the Hh signaling with PDE4 was investigated. Moreover, since Smo mutations occur at various stages of tumorigenesis, efficient inhibitors against the signaling components downstream of Smo are required for efficient therapy. In accordance, Western blot analysis of these key markers revealed that rolipram treatment reduced the levels of glioma-associated oncogene homolog 1 (GLI1) in both MDA-MB-231 and MCF-7 cell lines, in a time-dependent manner. In contrast, the level of glioma-associated oncogene homolog 2 repressor (GLI2R) increased with time in MCF-7 cells, whereas, in case of MDA-MB-231 cells, GLI2R increased up to 24 h of rolipram treatment but decreased slightly at 48 h of treatment. On the other hand, the level of GLI2 full length (GLI2FL) protein remained unchanged with respect to time in the case of MCF-7 cells (Fig. 2*A*) but significantly decreased with time in the case of MDA-MB-231 cells (Fig. 2*B*). Moreover, the level of glioma-associated oncogene homolog 3 repressor (GLI3R), which also acts as repressor of Hh signaling pathway, increased with time in both hormone-responsive and TNBC cells. Simultaneously, the levels of GLI proteins in MCF-7 and MDA-MB-231 cells were found to be unaltered in response to cotreatment with H89 (10 μM) and rolipram for 24 h, compared with control cells and cells treated only with 10 μM of H89 for 24 h (Figs. 2, *A* and *B*, and S1).

To ascertain if rolipram is able to induce ubiquitination of GLI2FL and GLI3 full length (GLI3FL), antibodies were raised against the regions of the proteins, containing amino acids from 991 to 1008 of translated region of GLI2FL (Fig. S2, *A* and *B*) and from 1030 to 1045 amino acids of translated region of GLI3FL (Fig. S3, *A* and *B*). These selected portions do not reside within the repressor form of the proteins and was therefore used for analyzing ubiquitination (13, 14). The results depicted that significant ubiquitination of GLI2 and GLI3 occurred at different time points, ranging from 6 h to 24 h, after rolipram treatment in MCF-7 cells. Similarly, ubiquitination of both proteins was also observed in MDA-MB-231 cells (Fig. 2, *C* and *D*). Interestingly, as described in Fig. 2*A*, the intracellular levels of GLI2FL do not decrease in MCF-7 cells with time-dependent rolipram treatment; however, the ubiquitination of the GLI2 (Fig. 2*C*) and the expression of GLI2R (Fig. 2*A*) in MCF-7 cells increased significantly with time-dependent rolipram treatment. Initially, proteins from both MCF-7 and MDA-MB-231 cell lines, which were not transfected with HA-Ub plasmid, were also assessed for ubiquitination after rolipram treatment to check the specificity of the plasmid (Fig. S4, *A* and *B*). Moreover, rolipram was not able to induce ubiquitination of GLI2 and GLI3 in both the cell lines in the presence of H89 (Fig. S3). In addition, it was also observed that rolipram, in combination with TAK-243 (0.1 μM), an inhibitor of ubiquitin-activating enzyme (UAE), failed to produce the repressor forms of both GLI2 and GLI3 (Fig. S4*D*).

### Rolipram recruits GSK3**β** to augment the PKA-mediated ubiquitination of GLI2FL and GLI3FL

Since it was evident that rolipram induced the ubiquitination of GLI2FL and GLI3FL to form GLI2R and GLI3R, the status of GSK3β was investigated. While looking for the modulators of GSK3β and their possible interactions with PKA, the levels of GSK3β and its upstream regulators were determined by Western blot analyses in both MCF-7 and MDA-MB-231 cells in a time-dependent manner. In accordance to temporal ubiquitination of GLI2 and GLI3, it was observed that rolipram treatment led to increased levels of PKA and induced elevated expression of phosphatase and TENsin homolog (PTEN) by threefold within 12 h. Subsequently, the levels of phospho-Akt were also observed to be reduced in both the cell lines, which might be indicative of the negative regulation of PTEN on the activity of PI3K. In addition, reduced levels of phosphorylated-GSK3β were also observed on rolipram treatment (Fig. 2, *E* and *F*).

### Rolipram retarded wound healing and reduced cell migration in breast cancer cells

**Figure 3:**
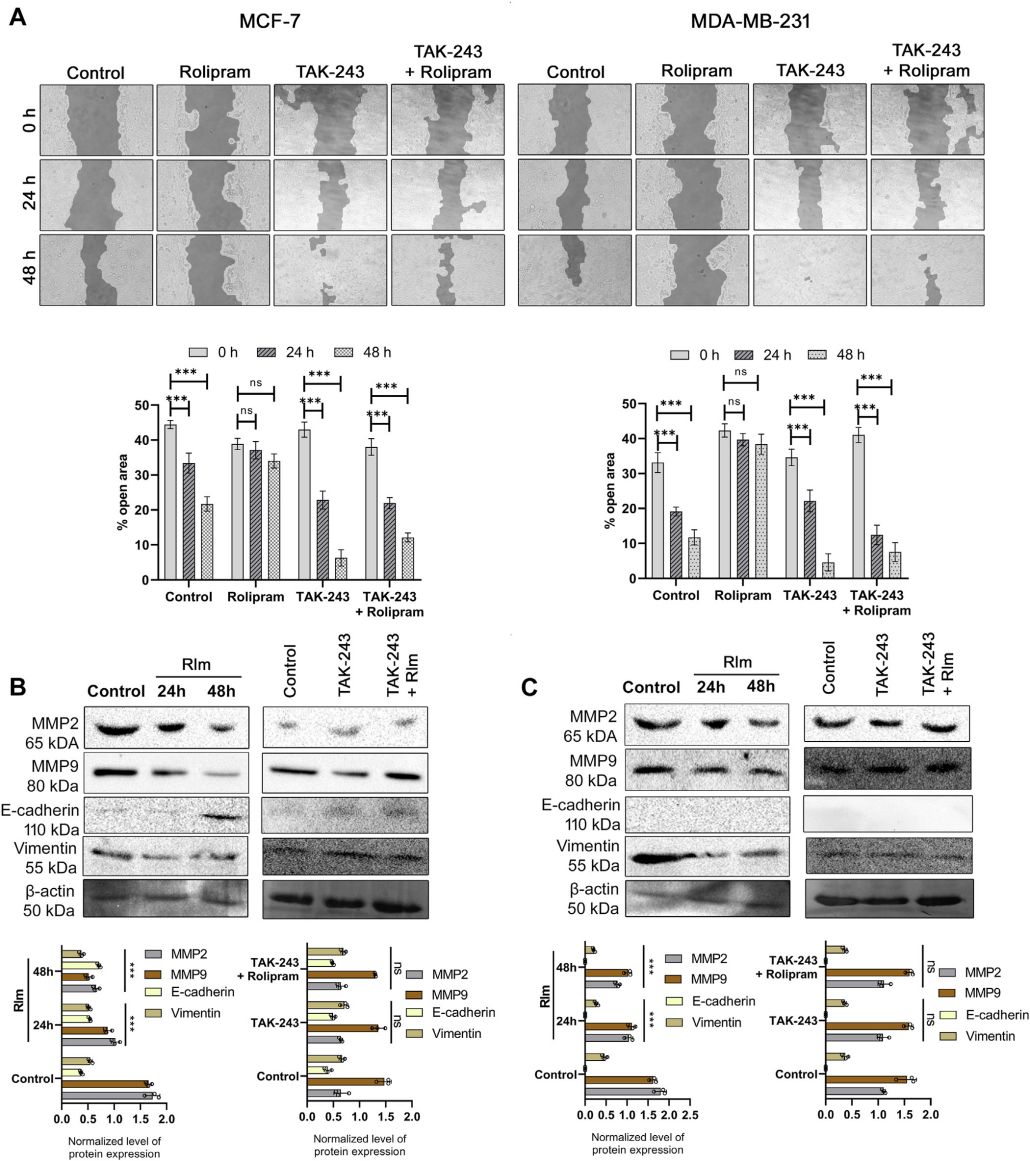
**Effect of rolipram on cell migration**. *A*, microscopic images showing status of mechanical wounds and comparison of wound closure at 24 and 48 h for untreated control MCF-7 and MDA-MB-231 cells and cells treated with rolipram at its IC_50_ doses. Corresponding bar plots represent the percentage of open area obtained analyzing the microscopic images with Tscratch software. Each set was repeated at least three times independently (∗∗∗*p* < 0.001 and ns [not significant] indicates *p* > 0.05). *C*, expression of MMP2, MMP9, E-cadherin, and vimentin in untreated and rolipram-treated MCF-7 cells (*B*) and MDA-MB-231 cells (*C*) at 24 and 48 h. The protein expressions were normalized against β-tubulin. MMP, matrix metalloproteinase.

It was next determined how modulation of Hh signaling by rolipram affects the migration potential of the breast cancer cell lines. The data revealed that untreated MCF-7 cells had 45% of open area, corresponding to the mechanical wound, which reduced to 34% by 24 h and 22% by 48 h (*p* < 0.001). However, rolipram-treated MCF-7 cells failed to significantly reduce the percentage of open area, even after 48 h. Similarly, in case of MDA-MB-231 cells, the untreated cells were able to reduce the percentage of open area from 33% to 20% by 24 h and to 12% by 48 h, respectively (*p* < 0.001), whereas rolipram-treated cells showed no significant change in the percentage of open area with time (Fig. 3*A*). Rolipram failed to retard the cell migration capability in both the cell lines in the presence of TAK-243 (0.1 μM) (Fig. 3*A*). Since the elevated levels of several isoforms of matrix metalloproteinases (MMPs) and altered levels of E-cadherin and vimentin present in the cancer microenvironment can directly induce cell migration and wound healing, Western blot analysis of the expression of MMPs, E-cadherin, and vimentin revealed that rolipram was effective in downregulating the expression of MMP2 by twofold in both the breast cancer cells. Similarly, MMP9 expression reduced by fourfold in hormone-responsive cells in 48 h, whereas in TNBC cells, twofold reduction was evident. The level of E-cadherin was elevated by twofold within 24 h of rolipram treatment in MCF-7 cells, whereas no conclusive change was observed after 24 h of treatment on MDA-MB-231 cells. Concomitant decrease in the levels of vimentin was also observed in MCF-7 and MDA-MB-231 cells after 48 h of rolipram treatment (Fig. 3, *B* and *C*). In contrast, rolipram, in the presence of TAK-243, failed to exert any of the aforementioned effects on the expressions of MMPs, E-cadherin, and vimentin in both cell lines (Fig. 3, *B* and *C*).

### Rolipram regressed tumor growth in mice

**Figure 4:**
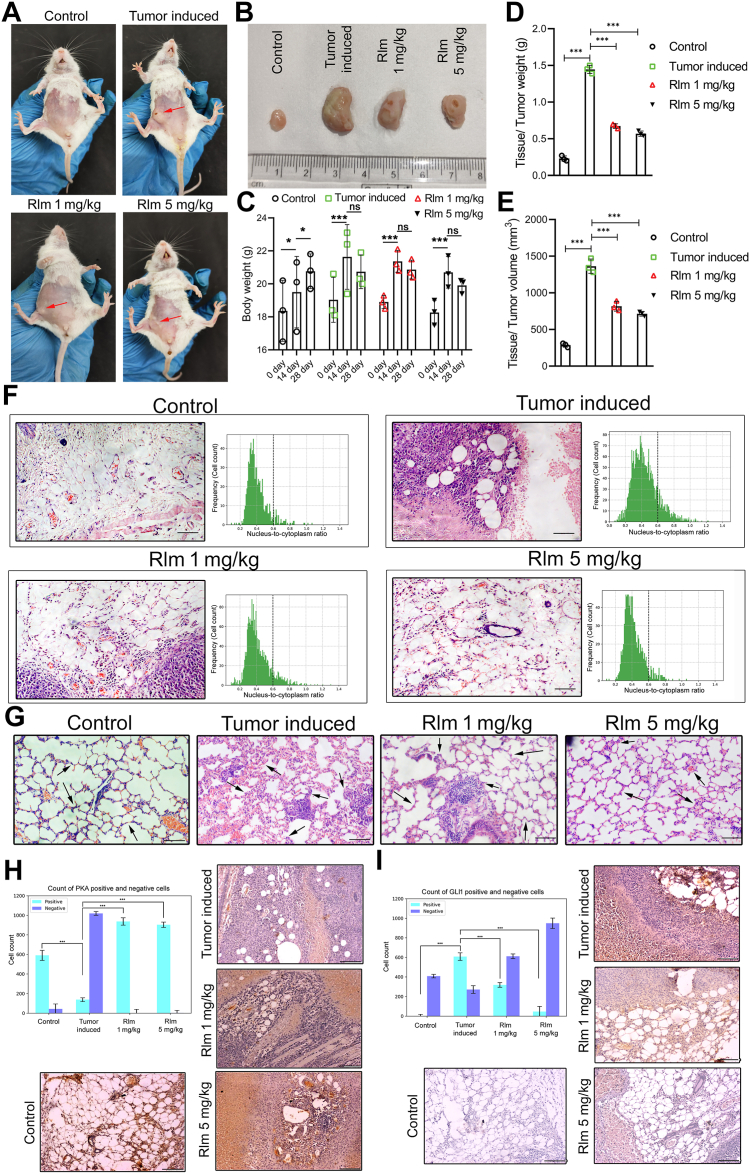
**Effect of rolipram on tumor-bearing mice model.***A*, images of healthy control mice, mice with induced solid tumor, and tumor-bearing mice treated with 1 mg/kg bodyweight and 5 mg/kg bodyweight of rolipram. *B*, images of the normal mammary gland of mice, tumor developed at mammary fat pad, and tumors after treatment with rolipram at 1 mg/kg bodyweight and 5 mg/kg bodyweight, respectively. *C*, comparison of bodyweight of healthy control mice, tumor-bearing mice, and tumor-bearing mice treated with rolipram at 1 and 5 mg/kg of bodyweight on day 0, day 14, and day 28. Comparison of weight (*D*) and volume (*E*) of mammary tissue of control mice with tumor-bearing mice and tumor-bearing mice treated with rolipram at 1 and 5 mg/kg of bodyweight, respectively (∗∗∗*p* < 0.001). *F*, histological analysis of mammary tissue of healthy control mice, untreated tumor-bearing mice, and tumor-bearing mice treated with rolipram at 1 and 5 mg/kg of bodyweight respectively, and the corresponding histogram showing frequency (cell count) of nucleus-to-cytoplasm ratio of cells. *G*, histological analysis of lung of control mice, tumor-induced mice, and rolipram-treated tumor-induced mice. Representative immunohistochemistry images and corresponding bar plots of DAB-positive and DAB-negative cells showing the expression of PKA (*H*) and GLI1 (*I*) in control (mammary fat pad from healthy mice), untreated mammary tumors, and mammary tumors treated with either 1 mg/kg bodyweight or 5 mg/kg bodyweight rolipram, respectively. Brown precipitates, indicated by *arrows*, represent positive staining for PKA or GLI1. All experiments were repeated at least three times. Magnification: 10X; scale bar represents 100 μm. DAB, 3,3′-diaminobenzidine; GLI1, glioma-associated oncogene homolog 1.

Since rolipram was found to be effective in *in vitro* assays in breast cancer cells, we delineated whether rolipram can exert similar antiproliferative effects *in vivo*. Fig. 4*A* depicts the mammary tumor (*red arrow*) and its significant regression in rolipram-treated mice. The tumors harvested from different groups of mice also depicted significant reduction in tumor size on rolipram treatment (Fig. 4*B*). Comparison of the bodyweights on day 0, day 14, and day 28 of each mouse from the four groups showed that although the normal mice gained weight with time, the tumor-bearing mice gained twice as much weight gained by normal mice by day 14 (*p* < 0.001) (Fig. 4*C*). No significant changes in the bodyweight of tumor-bearing mice were observed after 1 mg/kg bodyweight or 5 mg/kg bodyweight of rolipram treatment. Fig. 4, *D* and *E* depicted that both the weight and volume of the tumor in mice reduced by twofold after 14 days of treatment with 1 mg/kg bodyweight of rolipram. On the other hand, treatment with 5 mg/kg bodyweight of rolipram reduced tumor weight by 3.4-fold and tumor volume by 2.8-fold, respectively (*p* < 0.001).

Histological analysis of the normal mouse mammary gland revealed mammary ducts containing epithelial cells and the stroma comprising mammary fat pad and fibrous connective tissues. Histoarchitecture of tumor-bearing mice revealed accumulation of multinucleated cells in proximity of the glandular region interspersed within the mammary fat pad. Significant loss of structural integrity of the mammary fat pad was also evident in tumor-bearing mice. Rolipram treatment, on the other hand, substantially (*p* < 0.001) reduced multinucleated cells in the mammary fat pad and glandular region of mice, along with restoration of the normal glandular structure. Moreover, the corresponding histogram plots of the nucleus-to-cytoplasm ratio of the cells clearly depicted that a significant number of cells were having higher nucleus-to-cytoplasm ratio in tumor-bearing mice compared with control mice. Interestingly, the number of cells with higher nucleus-to-cytoplasm ratio decreased gradually with increasing doses of rolipram (Fig. 4*F*). The histological analyses of lungs of mice revealed that the alveolar lining of lung significantly thickened in tumor-bearing mice compared with control mice, which might be indicative of cell infiltration or metastasis in the lung tissues of mice. Several packed clusters of more than four cells having abnormal nuclei were also observed in the lung of tumor-induced mice (pointed with *black arrows* in Fig. 4*G*). Rolipram treatment of mice was observed to not only significantly reduce metastatic cellular infiltration in the lung tissues of mice but also visibly reduced the number of cell clusters with abnormal nuclei. Interestingly, the histological appearance of the lung tissues restored toward normal lung histology in a dose-dependent manner (Fig. 4*G*). The quantification of the number of cells present in a unit area (480 px × 400 px) of histological sections revealed that the number of cells increased significantly in the lung tissue tumor-induced mice (213 ± 20) compared with control mice (143 ± 19). Rolipram treatment, on the other hand, significantly reduced the number of cells per unit area in a dose-dependent manner (154 ± 11 and 119 ± 10, respectively, in 1 and 5 mg/kg bodyweight rolipram-treated groups) (Fig. S7*A*).

Next, the expressions of PKA and GLI1 were checked in the normal and tumor tissues. Immunohistochemistry depicted statistically significant reduction of PKA expression in the tumor tissues compared with normal mammary glands of mice (*p* < 0.001). Analysis of the microscopic images with QuPath software revealed that there was more than threefold decrease in the PKA-positive cells in the tumor tissue of tumor-bearing mice compared with mammary fat pad of control mice. Simultaneously, the PKA-positive cells increased by nearly 1.5-fold (*p* < 0.001) when treated with 1 and 5 mg/kg bodyweight of rolipram compared with tumor tissues (Figs. 4*H*, S5). In contrast, there was no significant expression of GLI1 observed in the mammary tissues of control mice, whereas significantly increased expression of the same was evident in the tumor tissue of the tumor-bearing mice. Concomitant reduction of GLI1 expression was observed in rolipram-treated mice (*p* < 0.001), indicating inhibition of the Hh signaling pathway. The GLI1 expression in tumor tissue of tumor-bearing mice reduced by twofold, when treated with 1 mg/kg bodyweight of rolipram, and by sixfold, when treated with 5 mg/kg bodyweight of rolipram (Figs. 4*I*, S6). Similar trend of expressions of PKA and GLI1 was also confirmed by Western blotting from breast tissue sample from each group of mice (Fig. S7*B*).

### Effect of rolipram on hepatorenal histopathology and biochemistry

**Figure 5:**
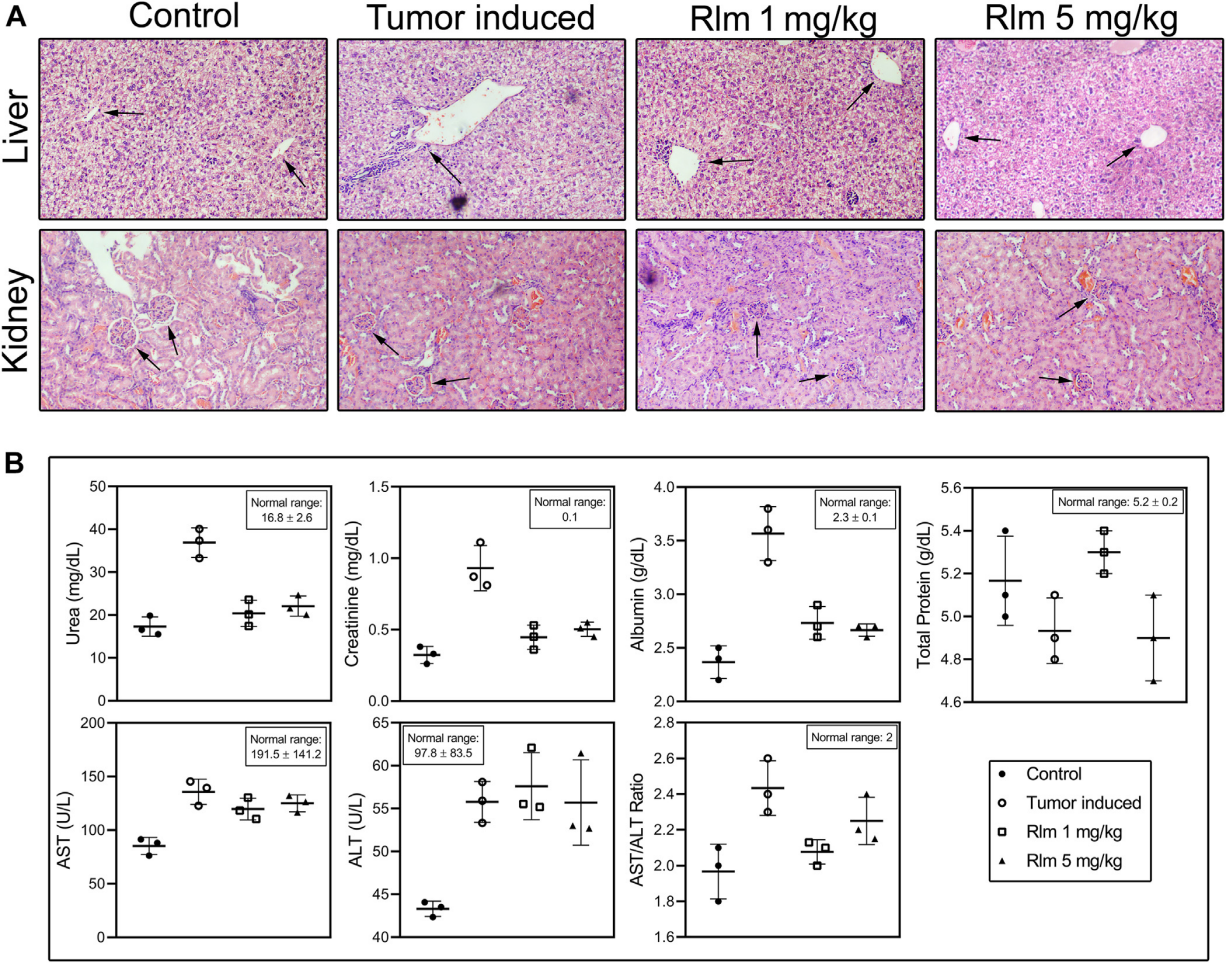
**Hepatorenal histopathology and biochemistry of mice**. *A*, histological analysis of liver and kidney of healthy control mice, tumor-induced mice, and tumor-induced mice treated with rolipram at 1 and 5 mg/kg of bodyweight, respectively. In liver sections, the *black arrows* point the hepatic central veins, and in kidney sections, the *black arrows* point glomerulus. *B*, graphical representations of levels of serum biochemical parameters corresponding to liver and kidney functions of mice.

Finally, the effect of dose-dependent rolipram treatment was evaluated on vital organs of mice, such as the liver and kidney. Hepatic vein dilation was observed in tumor-bearing mice. Such dilation was not evident in rolipram-treated tumor-bearing mice compared with normal mice (Fig. 5*A*). The analyses of biochemical parameters in different groups of mice demonstrated significantly higher aspartate aminotransferase (AST) and alanine aminotransferase (ALT) levels in tumor-bearing mice compared with normal mice (*p* < 0.001). The values reduced with statistical significance following rolipram treatment (*p* < 0.001). Similarly, total protein and serum albumin levels were modulated in tumor-bearing mice. Serum creatinine and urea, the two important markers to assess kidney function, were observed to increase by twofold in tumor-bearing mice (*p* < 0.001) (Table 1, Fig. 5*B*). Rolipram treatment reduced the levels close to the values in the control group (*p* < 0.001).

**Table 1:** Levels and activities of serum biochemical parameters corresponding to kidney and liver functions of healthy control mice, tumor-induced mice, and tumor-induced mice treated with rolipram at 1 and 5 mg/kg of bodyweight

Groups	Kidney function		Liver function			
	Urea (mg/dl)	Creatinine (mg/dl)	AST (U/L)	ALT (U/L)	Albumin (g/dl)	Protein (g/dl)
Normal	17.30 ± 2.25	0.32 ± 0.06	85.23 ± 7.97	43.31 ± 0.88	2.36 ± 0.15	5.16 ± 0.20
Tumor	36.89 ± 3.43	0.93 ± 0.15	135.85 ± 11.7	55.77 ± 2.38	3.56 ± 0.25	4.93 ± 0.15
Tumor + rolipram (1 mg/kg)	20.39 ± 3.06	0.45 ± 0.08	119.69 ± 10.1	57.60 ± 3.89	2.73 ± 0.15	5.30 ± 0.10
Tumor + rolipram (5 mg/kg)	22.08 ± 2.34	0.50 ± 0.05	33.48 ± 7.86	55.69 ± 4.97	2.66 ± 0.05	4.90 ± 0.20

Data are expressed as mean ± SD (n = 3).

## Discussion

Breast cancer is a serious problem worldwide because of its association with tumor relapse and chemoresistance (15, 16). Among the different types of breast cancer, treating TNBC is more challenging because of its heterogeneity, association with a significant population of brCSCs, and lack of predictive biomarkers providing effective targeted therapies. In a previous study conducted by our group, we showed that rolipram drives cell death *via* PKA–PI3K–Akt–mTOR (mammalian target of rapamycin) axis in TNBCs, whereas in case of brCSCs, we observed noncanonical activation of a pathway directly influencing PKA and mTOR, bypassing PI3K and Akt (11). Researchers have indicated that the cAMP–PKA axis is also directly associated with Hh signaling (3). Therefore, the mechanism of action of rolipram in both endoplasmic reticulum/progesterone receptor–positive breast cancer and TNBCs, and the crosstalk between PKA and Hh, was explored in this study.

Hh signaling pathway mediated by Smo and GLI proteins is a well-known pathway that is deregulated in several cancers, including breast cancer (2, 17, 18). Hh signaling is associated with progression and aggressive metastasis of breast cancer (18, 19). Studies have conclusively stated that increased PI3K–Akt–mTOR activity is associated with enhanced Hh signaling through positive regulation of GLI1 transcription factors and downregulation of GSK3β and AMP-activated protein kinase (20). In contrast, PKA induces GLI2–GLI3 processing by multisite phosphorylation at C terminus of GLI2–GLI3 recognized subsequently by E3 ubiquitin ligase, finally degraded by proteasome (21). Therefore, considering the important roles of Hh pathway, targeting the same with existing and newly discovered Hh inhibitors gained a significant importance as far as exploring anticancer agents is concerned (22). Several Hh inhibitors, especially the Smo targets like vismodegib and soridegib (both Food and Drug Administration–approved Hh inhibitors), have several advantages. Smo has structural similarities with G-protein coupled receptor opening the possibility to have multiple drug targets. Unfortunately, Smo inhibitors of Hh pathway, though have promising anticancer potential, have shown substantial resistance, especially in breast cancer (22). The one major cause of this resistance was genetic mutation of Smo leading to increased tumor relapse causing the failure of the first-generation Smo-targeted drugs (23). Hence, exploration of novel anticancer agents inhibiting Hh pathway downstream of Smo *via* the transcriptional regulation of GLI proteins has become the need of the hour.

Our study deciphered that PDE4 inhibitor, rolipram, not only increased intracellular PKA by cAMP-dependent manner but also reduced the inhibitory effect on GSK3β by reducing the chance of inhibitory phosphorylation of the same by PI3K–Akt pathway. The ubiquitination of the GLI2FL and GLI3FL to their respective repressor forms, GLI2R and GLI3R, because of rolipram treatment might be indicative of the fact that rolipram was able to augment the formation of PKA–CK1–GSK3β complex, which phosphorylates GLI2FL and GLI3FL at specific sites to initiate ubiquitin-mediated proteosomal degradation. Moreover, rolipram failed to induce ubiquitination and the formation of GLI2R and GLI3R both in the presence of H89, a PKA inhibitor, and TAK-243, an UAE inhibitor. These outcomes also substantiate the fact that rolipram treatment resulted in the formation of GLI2R and GLI3R as a result of PKA-mediated ubiquitination of the full-length forms of GLI2 and GLI3. Rolipram was found to be efficient in inhibiting the transcription factors of Hh signaling downstream of Smo, making it more effective than the conventional Hh inhibitors as Smo mutations would fail to make the inhibitor ineffective. Rolipram was also observed to be effective in BALB/c mice to regress TNBC. It was evident that rolipram could significantly reduce tumor volume and weight and also was effective in stopping cell infiltration in lung tissue. Although rolipram was able to alter the cell migration capabilities of hormone-responsive and human TNBC cell lines, results obtained for the expression of MMP2/9 failed to show conclusive downregulation by rolipram, the triple-negative cell line. Further investigation is required to study the activities of MMP2/9 in this context. It is also important to understand whether the modulation of the expressions and activities of MMP2/9 is a PKA-dependent event or a rolipram has off-targets like many other drugs available in the market (24). To conclude, it can be said that antiproliferative, proapoptotic, and anti-invasive consequences of rolipram on breast cancer cell lines and mice tissue seemed to be mediated *via* cAMP–PKA-mediated Hh signaling modulation with the repression actions of GLI2 and GLI3 along with the cAMP–PKA–mTOR signaling pathway. Though further studies are required to understand how rolipram modulates the different extracellular matrix molecules, whether the modulations are solely cAMP–PKA mediated or there are other interesting mechanisms. Hence, rolipram can replace any traditional Smo inhibitors to bypass any drug resistance caused by Smo mutations. Further studies are needed to understand how rolipram modulates Hh signaling in brCSCs and whether it is effective against chemoresistance, invasion, and metastasis. Rolipram served as a prototype molecule as a potential antidepressant and showed efficacy against Alzheimer’s disease after clearing clinical trial (10). It was discontinued as the drug had a narrow window to show its therapeutic actions without gastrointestinal side effects (25). Keeping all these facts in mind, our data might encourage designing clinical trials using rolipram in combination with existing chemotherapeutic drugs, such as paclitaxel, to determine the effective and tolerable doses of rolipram in patient with breast cancer, thereby repurposing the same for breast cancer. This might be an effective method of having a drug action against the brCSCs along with the cancer cells decreasing the chances of chemoresistance against frequently used drugs like paclitaxel at high doses.

## Experimental procedures

### Ethics statement

The study with laboratory animals was in strict accordance with the guidelines for the Care and Use of Laboratory Animals from the Committee for the Purpose of Control and Supervision of the Experiment on Animals (CPCSEA). The protocols used in this study were approved by the Institutional Animal Ethics Committee on Animal Experiments of the University of Kalyani (892/GO/Re/S/01/CPCSEA), Kalyani, Nadia, West Bengal, India and University of Calcutta (registration number: 885/ac/05/CPCSEA).

### Materials

Rolipram (a PDE4 inhibitor) was purchased from Sigma–Aldrich (catalog no.: R6520) and dissolved in ethanol to prepare a stock solution of 10 mM. The selective PKA inhibitor H89 was also procured from Sigma–Aldrich (catalog no.: B1427) and dissolved in dimethyl sulfoxide (DMSO) to prepare a stock solution of 1 mM. Inhibitor of UAE, TAK-243, was obtained from Cayman Chemical Company (catalog no.: 30108) and dissolved in DMSO to prepare a stock solution of 100 μM. The primary antibodies used in this study are PKA (Santa Cruz Biotechnology; catalog no.: sc-28315, Research Resource Identifier [RRID]: AB_628136), PTEN (Cell Signaling Technology; catalog no.: 9552, RRID: AB_10694066), PI3K (Cell Signaling Technology; catalog no.: 4249, RRID: AB_2165248), Akt (Cell Signaling Technology; catalog no.: 9272 [also 9272S], RRID: AB_329827), p-GSK3β (Santa Cruz Biotechnology; catalog no.: sc-373800, RRID: AB_10920410), GLI1 (Novus Biologicals; catalog no.: NB600-600, RRID: AB_2111758), GLI2 (Novus Biologicals; catalog no.: NB600-874, RRID: AB_10001953), GLI3 (Novus Biologicals; catalog no.: NBP2-29627), anti-HA (Cell Signaling Technology; catalog no.: 2367, RRID: AB_10691311), MMP2 (Cell Signaling Technology; catalog no.: 4022, RRID: AB_2266622), MMP9 (Cell Signaling Technology; catalog no.: 3852, RRID: AB_2144868), E-cadherin (Cell Signaling Technology; catalog no.: 14472, RRID: AB_2728770), and vimentin (Cell Signaling Technology; catalog no.: 3932 [also 3932S], RRID: AB_2288553). The secondary antibodies used in this study are goat antimouse IgG–horseradish peroxidase (HRP) (Santa Cruz Biotechnology; catalog no.: sc-2005, RRID: AB_631736) and goat anti-rabbit IgG-HRP (Santa Cruz Biotechnology; catalog no.: sc-2004, RRID: AB_631746).

### Cell lines and model organisms

Two human breast cancer cell lines, MCF-7 (RRID: CVCL_0031) and MDA-MB-231 (RRID: CVCL_0062), were used for this study. The mouse (*Mus musculus*) breast carcinoma cell line 4T1 (RRID: CVCL_0125) was also used in this study. The cell lines were procured from the National Centre for Cell Science, Pune, India. The female BALB/c mice (bodyweight of 15–20 g before commencement of experiments) and female New Zealand albino rabbits (bodyweight of 4–4.5 kg before commencement of experiments) were purchased from Centre for Laboratory Animal Research and Training (CLART), West Bengal Livestock Development Corporation Ltd (A Government of West Bengal undertaking), Kalyani, Nadia, West Bengal, India.

### Cell culture

The cells were maintained at 37°C in a humidified 5% CO_2_ environment in Dulbecco's modified Eagle's medium (Gibco; catalog no.: 11885084), supplemented with 10% fetal bovine serum (Gibco; catalog no.: 16140071) and 100 U/ml penicillin–streptomycin (Gibco; catalog no.: 15140122). All the cells were tested for the presence of any contaminants like *Mycoplasma* before starting an experiment. Cells in their log phase were used for all subsequent experiments (26).

### Cell viability assay

To determine the concentration of rolipram required for 50% growth inhibition (IC_50_) of MCF-7 and MDA-MB-231 cells, MTT (3-[4,5-dimethylthiazol-2-yl]-2,5 diphenyl tetrazolium bromide) assay, which relies on the reduction of MTT to form purple-colored formazan crystals by mitochondrial dehydrogenases, was performed (27). Cells were seeded in a 96-well plate at a density of 3 × 10^3^ cells/well and treated with increasing concentrations of rolipram ranging from 1 to 80 μM. MTT (G Biosciences; catalog no.: RC1130) was added after 24 h of treatment, formazan crystals were dissolved in 100 μl of DMSO, and absorbance measured at 595 nm using the BioRad iMark Microplate Reader (RRID: SCR_023799). The percentage of metabolically active cells was calculated with respect to untreated control cells, negating the blank reading. Graphs were plotted for the percentages of viable cells against the concentrations of rolipram to obtain linear trend-line equations, which were used to derive the IC_50_ concentration of rolipram for each cell lines. The IC_50_ doses were used for all subsequent experiments (28).

### cAMP assay

To determine the intracellular level of cAMP, a sandwich ELISA-based cAMP assay kit was used, which relies on preparation of a standard curve for cAMP (pmol/ml) and denoting all the experimental values from the standard curve, and finally expressing as mean values with standard errors of triplicate samples per treatment group. The assay kit was obtained from Cayman Chemicals (item no.: 581001), and the assay was performed following the manufacturer’s protocol after washing the cells with 1X PBS (composition: 137 mM NaCl, 2.7 mM KCl, 10 mM Na_2_HPO_4_, and 1.8 mM KH_2_PO_4_) and treating with 0.1 M HCl for 20 min and sonicating the cells for lysis. In brief, the cell lysate was added to the 96-well plate precoated with mouse monoclonal anti-rabbit IgG and which also contained cAMP–acetylcholinesterase (cAMP tracer) that competes with the free cAMP in the cell lysate to bind to the cAMP-specific rabbit antibody. Following several washes, the absorbance of the color generated because of addition of acetylcholinesterase substrate is measured at 412 nm by Bio-Rad iMark Microplate Reader (RRID: SCR_023799), which is inversely proportional to the amount of free cAMP present in the cell lysate (29).

### Analysis of DNA content of cells

After treating the cells with rolipram for 24 h, cells were stained with PI (BD Pharmingen PI/RNase Staining Buffer; BD Biosciences; catalog no.: BD550825), following fixing of cells with 70% ethanol for 1 h at 4°C and permeabilization with 0.1% Triton X-100 (Sigma–Aldrich; catalog no.: X100). The cells were then analyzed using BD LSRFortessa flow cytometer (RRID: SCR_019600), and the data were analyzed using BD FACSDiva 9.0 software (30) (RRID: SCR_001456).

### Double staining of cells with Annexin V-FITC and PI

To analyze if the cells have undergone necrosis or apoptosis because of 24 h of rolipram treatment, cells were double stained with Annexin V-FITC and PI using BD Pharmingen Annexin V:FITC Apoptosis Detection Kit (BD Biosciences; catalog no.: BD556547) using the manufacturer’s protocol. The data obtained from BD LSRFortessa flow cytometer (RRID: SCR_019600) were analyzed using BD FACSDiva 9.0 software (31, 32) (RRID: SCR_001456).

### Raising of antibodies

Specific peptides, HNVNPGPLPPCADRRGLR and CNPPAMATSAEKRSLV, corresponding to the transactivation domains of GLI2 and GLI3, respectively, were designed with the help of ExPASy Bioinformatics Resource Portal (RRID: SCR_012880) and synthesized (Figs. S2 and 3, *A* and *B*). These peptides are then emulsified in Freund’s complete adjuvant (Sigma–Aldrich; catalog no.: F5881) before injecting it subcutaneously in New Zealand albino rabbits. The sites were cleaned and disinfected with alcohol and betadine 10% solution (Win-Medicare Pvt Ltd). After 15 days of administration of the first dose, the second dose of the peptides, emulsified in Freund’s incomplete adjuvant (Sigma–Aldrich; catalog no.: F5506), was injected subcutaneously. After 15 days of the second dose, blood was collected from the rabbits and antibodies were purified from the serum using AminoLink Immobilization Kit (Thermo Scientific; catalog no.: 44890) following the manufacturer’s protocol (33). The specificity of the antibodies against the desired targets was tested with different amount of protein lysates from MCF-7 and MDA-MB-231 cell lines (see Figs. S2*C* and 3*C* for raised antibodies against GLI2 and GLI3, respectively).

### Western blot analysis

After treatment of cells with rolipram, proteins were extracted from cells by lysing the cells with radioimmunoprecipitation assay buffer, comprising of 150 mM NaCl, 1% Triton X-100, 0.5% sodium deoxycholate, and 0.1% SDS. Equal amount of proteins (40 μg) were resolved using SDS-PAGE before transferring onto nitrocellulose membrane (Immobilon-NC Transfer Membrane; Merck Millipore; catalog no.: HATF00010). The membranes were then incubated overnight with primary antibody at a dilution of 1:1000 at 4°C followed by incubation with secondary antibodies at a dilution of 1:5000 for 2 h at room temperature. Immune detection was performed using femtoLUCENT PLUS-HRP (G-Biosciences; catalog no.: 786-003), and the image was obtained from the Bio-Rad ChemiDoc XRS+ system. Analysis of protein expression was carried out using Bio-Rad Image Lab software (RRID: SCR_014210) and National Institutes of Health ImageJ software (34) (RRID: SCR_003070). All protein expressions were normalized against β-tubulin (loading control).

### Study of ubiquitination of protein

HA-Ubiquitin plasmid (Addgene; catalog no.: plasmid #18712, RRID: Addgene_18712) was transfected into MCF-7 and MDA-MB-231 cells using X-tremeGENE HP DNA Transfection Reagent (Roche; catalog no.: XTGHP-RO). Proteins were extracted from the untreated control cells and cells treated with rolipram for 24 h and were immunoprecipitated using antibodies raised against GLI2 and GLI3. The immunoprecipitation involved overnight incubation of protein and antibodies in Pierce Protein A/G Agarose (Thermo Scientific; catalog no.: 20421) at 4°C. Following immunoprecipitation, the precipitate was subjected to Western blot analysis using anti-HA antibodies (Cell Signaling Technology; catalog no.: 2367; RRID: AB_10691311) to observe ubiquitination of the desired proteins. Cells were cotreated with MG-132 (Sigma–Aldrich; catalog no.: M7449), a proteosomal blocker for ascertaining ubiquitination (35).

### Wound healing assay

Cells were seeded and allowed to attain a 2D monolayer of cells. An artificial wound was created using 100 μl pipette tip, and the healing procedure was observed in a time-dependent manner using an Olympus CKX53 Inverted Microscope (RRID: SCR_025025) at 10X magnification in untreated control cells and cells treated with rolipram (36). The percentage of open area was evaluated using Tscratch software (CSElab, Koumoutsakos group at ETH Zurich, https://github.com/cselab/TScratch) (RRID: SCR_014282).

### Solid tumor development in mice

Solid tumors were developed in BALB/c mice (n = 3) except the control group (group I) by injecting 1 × 10^4^ viable 4T1 (RRID: CVCL_0125) mice breast carcinoma cells (resuspended in 50 μl of PBS) into the inguinal fourth mammary fat pad, and the tumor was allowed to develop for 14 days (37, 38). Mice were randomly divided into three groups, *viz*., untreated tumor (group II), mice treated intraperitoneally with 1 mg//kg bodyweight of rolipram (group III), and mice receiving 5 mg/kg bodyweight of rolipram, intraperitoneally (group IV) (39, 40). After 14 days, the mice were sacrificed. Lung, kidney, liver, serum, and the tumors were collected for further experiments (15).

### Tumor weight and volume measurement

Weight of mammary fat pad of mice from group I and weight of tumors from groups II, III, and IV were measured using digital fine balance. Width and length of the tumors obtained from mice of groups II, III, and IV were measured using digital Vernier caliper. The volume was measured by the formula V = half × (W × W × L), where V is the volume, W is the width, and L is the length of the tumors (15, 41).

### Histological study

Tissue samples of mammary tissue, lung, kidney, and liver from each group of mice were fixed in Bouin’s solution (Sigma–Aldrich; catalog no.: HT10132), dehydrated in graded alcohol, and embedded in paraffin wax. Thin sections (5 μm) of tissues were mounted on glass slides and stained with hematoxylin (Sigma–Aldrich; catalog no.: H3136), following rehydration with graded alcohol. The samples were again dehydrated with graded alcohol and stained with eosin and mounted with Dystyrene Plasticizer Xylene (42). The images were obtained in an Olympus CKX53 Inverted Microscope (RRID: SCR_025025) at 10X magnifications with MagVision Software (Magnus). The QuPath (RRID: SCR_018257) software (43) was used to detect the cells and determine the area of individual cells and their corresponding nuclei in each image. The tab-delimited text file obtained from QuPath software was utilized by NumPy (44) (RRID: SCR_008633) to determine the nucleus-to-cytoplasm ratio of individual cells, and the frequency (cell count) of the nucleus-to-cytoplasm ratio was plotted by MatPlotLib (RRID: SCR_008624), a Python (RRID: SCR_008394) 2D plotting library. For quantifying the number of cells in a unit area of histological section of lung, each section image was divided into 12 equal parts (dimensions: 480 px × 400 px). The number of cells in each part was obtained from QuPath (RRID: SCR_018257) software, and the values were represented as mean ± SD.

### Immunohistochemistry of tumor tissue

Mice mammary fat pad and mammary tumor tissues were processed as for histology, and 5 μm-thick sections were mounted on glass slides. The tissue sections were deparaffinized with xylene and hydrated with ascending grades of alcohols (100% to 70%). Rehydrated sections were equilibrated in PBS. Antigen retrieval was performed by boiling the sections in a 0.1 M sodium citrate buffer (pH 6.0) for 10 min. Endogenous peroxidase activity was blocked by treating the sections with a 3% (v/v) hydrogen peroxide solution in PBS for 30 min. Nonspecific staining was prevented by incubating the sections with bovine serum albumin at room temperature for 1 h. Subsequently, the tissue sections were incubated with primary antibodies, namely anti-PKA (Santa Cruz Biotechnology; catalog no.: sc-28315) and anti-GLI1 (Novus Biologicals; catalog no.: NB600-600, RRID: AB_2111758) at a dilution of 1:100, for 18 h at 4°C. Next the sections were washed well with PBS and exposed to antimouse (Santa Cruz Biotechnology; catalog no.: sc-2005) and anti-rabbit (Santa Cruz Biotechnology; catalog no.: sc-2004) secondary antibodies, respectively, conjugated with HRP, at a dilution of 1:200 for 1 h at room temperature. The peroxidase activity was visualized using 3,3′-diaminobenzidine. The sections were counterstained with hematoxylin, and images were captured at 10X magnification in Olympus BX53 Microscope (45) (RRID: SCR_022568). The QuPath (RRID: SCR_018257) software (43) was used to analyze and detect the GLI1- and PKA-positive cells from the images. The frequency (cell count) of positive and negative cells as well as the mean absorbance of the positive cells were plotted using MatPlotLib (RRID: SCR_008624), a Python (RRID: SCR_008394) 2D plotting library.

### Analysis of serum biochemical parameters

The total protein in mouse serum was measured by biuret method, which relies on formation of reddish-violet color in alkaline copper solution (46). The absorptivity of the colored solution was measured at 570 nm and compared with known protein standards to determine the level of total protein in the serum. The serum urea level of mouse was determined using urea kit (Coral Clinical Systems; catalog no.: 1102240075), which relies on the reaction of phenolic chromogen and hypochlorite with the ammonia formed from urea (47). The absorbance of the test sample (AbsT) and standard sample (AbsS) was recorded at 570 nm, and the concentration of urea was determined by the formula urea (mg/dl) = (AbsT/AbsS) × 40, according to the manufacturer’s protocol. Level of serum creatinine was measured using creatinine kit (Coral Clinical Systems; catalog no.: 1101060035). Briefly, this kit utilizes the formation of a colored complex because of reaction between alkaline picric acid solution and creatinine (48). The initial absorbance of the test sample (Abs_1_T) and standard sample (Abs_1_S) was measured at 520 nm after 30 s of incubation, and the final absorbance of the test sample (Abs_2_T) and standard sample (Abs_2_S) was measured exactly after 60 s. The formula, creatinine (mg/dl) = (Abs_2_T – Abs_1_T)/(Abs_2_S – Abs_1_S) × 2, was used to determine the concentration of creatinine. Activities of AST and ALT were evaluated from the serum of mice using AST assay kit (catalog no.: MAK055) and ALT assay kit (catalog no.: MAK052) from Sigma–Aldrich, following the manufacturer’s protocol. The estimation of AST activity is based on the production of glutamate by the activity of AST present in the serum samples. The absorbance of the colored product was measured at 450 nm and compared with known standard samples to determine the activity (49). Similarly, the ALT assay kit is based on the production of pyruvate in the presence of ALT in serum. The activity of the ALT in mouse serum was determined by measuring the absorbance of the colored product at 570 nm and comparing with the known standard samples (50). All the spectrophotometric analyses were carried out in Bio-Rad iMark Microplate Reader (RRID: SCR_023799).

### Statistical analysis

All experiments were performed in triplicates. Data are presented as the mean values of “n” independent measurements, as indicated in the figure legends. All the statistical analyses were performed using GraphPad Prism (RRID: SCR_002798). Statistical comparisons between treated and untreated control groups were calculated by Student’s *t* test (two-tailed, independent), Mann–Whitney test, and analysis of variance, followed by Tukey's honestly significant difference test. *p* ≤ 0.05 was considered significant (51).

## Data availability

The data that support the findings of this study are contained within the article, and any additional information is available on request from the corresponding author.

## Supporting information

This article contains supporting information.

## Conflict of interest

The authors declare that they have no conflicts of interest with the contents of this article.
