# Supplementary material for: PDE4 inhibitor rolipram represses hedgehog signaling via ubiquitin-mediated proteolysis of GLI transcription factors to regress breast cancer
Source: J Biol Chem. 2025 Jan 27;301(3):108239. doi: 10.1016/j.jbc.2025.108239 (PMC11879692; doi:10.1016/j.jbc.2025.108239)
Supplement: Supporting Information [file mmc1.docx]

**Supporting Information**

**Figure S1:**


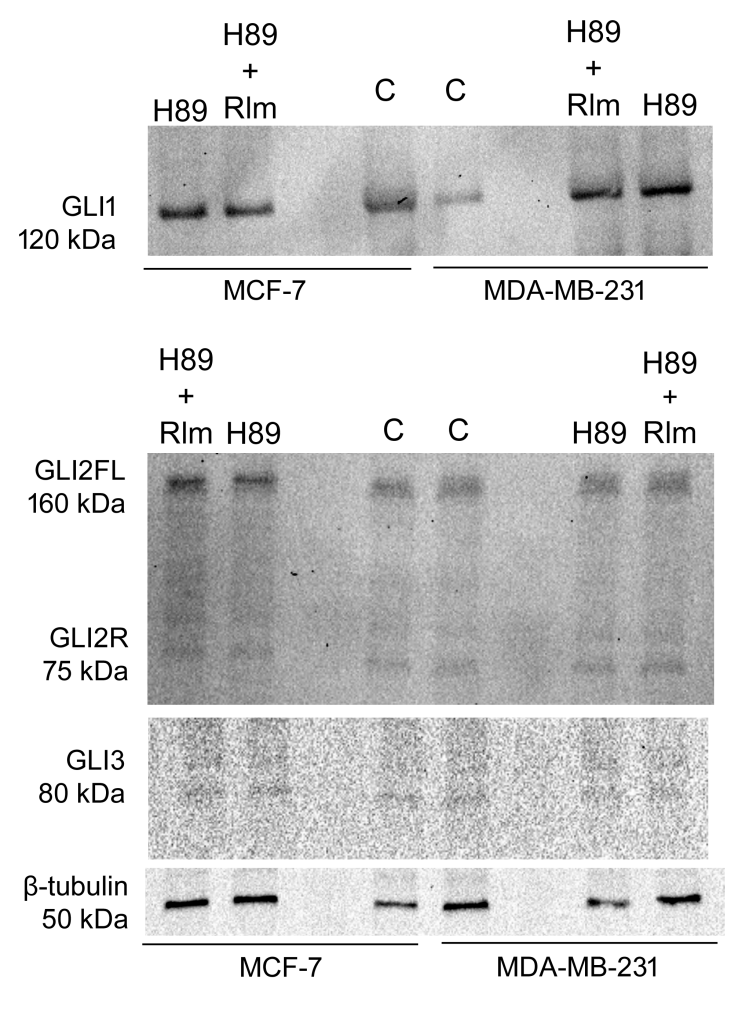


**Figure S1:** Expression of GLI1, GLI2FL, GLI2R, GLI3R in MCF-7 cells and MDA-MB-231 cells following H89 treatment and H89 + rolipram co-treatment for 24 hours.

**Figure S2:**


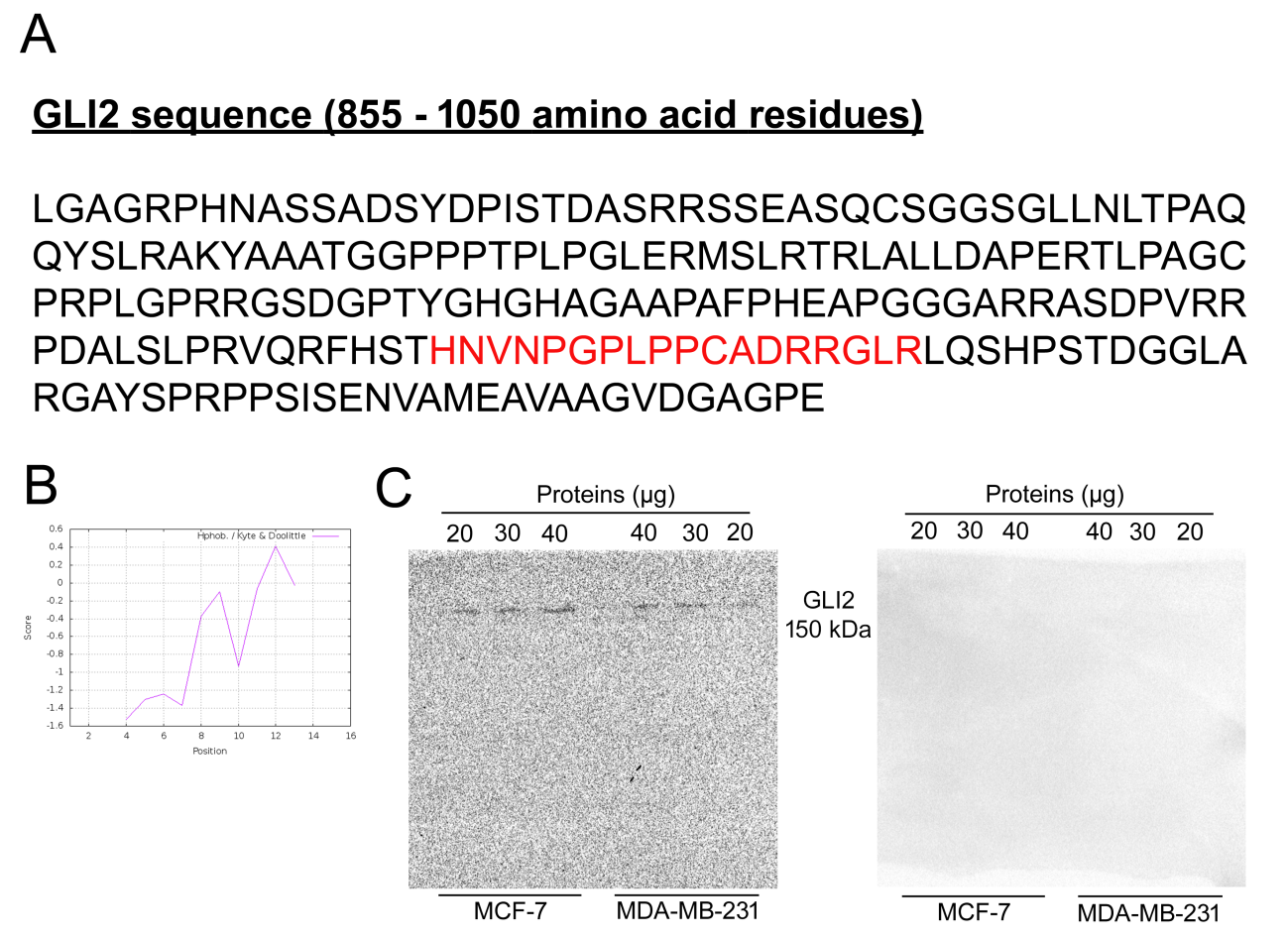


**Figure S2:** (A) Sequence of GLI2 ranging from 855 to 1050 amino acid residues. The red highlighted text represents the sequence of amino acid residues used for peptide synthesis for raising of antibody against GLI2. (B) The Kyte & Doolittle plot of the peptide sequence which corresponds to the hydrophobicity of the peptide sequence. (C) Western blot analysis of different amounts of protein from MCF-7, and MDA-MB-231 cell lines by raised anti-GLI3 antibody (left panel) and raised antibody incubated with the GLI2 peptide, used for raising the antibody (right panel).

**Figure S3:**


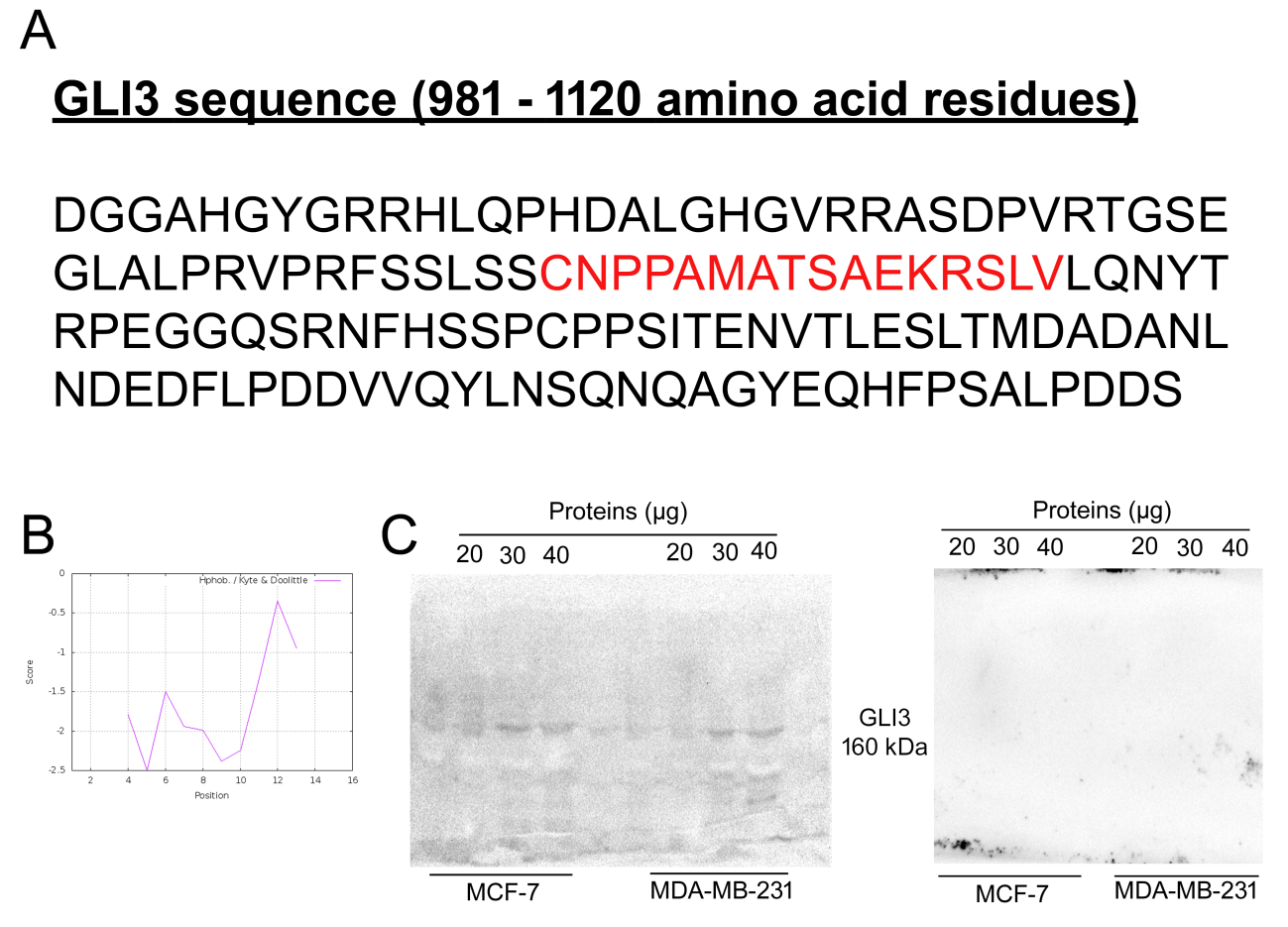


**Figure S3:** (A) Sequence of GLI3 ranging from 991 to 1008 amino acid residues. The red highlighted text represents the sequence of amino acid residues used for peptide synthesis for raising of antibody against GLI3. (B) The Kyte & Doolittle plot of the peptide sequence which corresponds to the hydrophobicity of the peptide sequence. (C) Western blot analysis of different amounts of protein from MCF-7, and MDA-MB-231 cell lines by raised anti-GLI3 antibody (left panel) and raised antibody incubated with the GLI3 peptide, used for raising the antibody (right panel).

**Figure S4:**


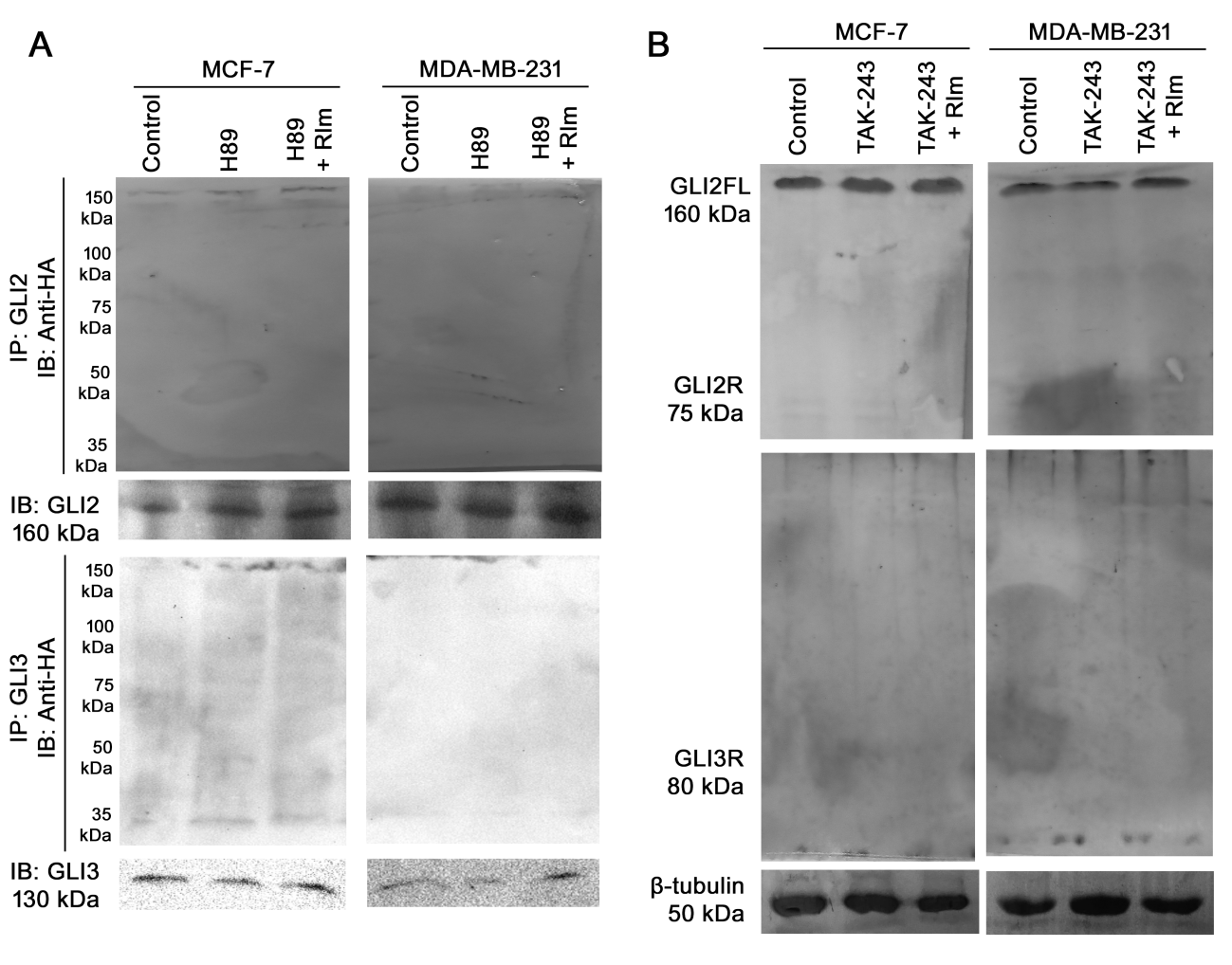


**Figure S4:** Co-imunoprecipitation in non-transfected MCF-7 cells and MDA-MB-231 cells (without HA-Ub plasmid) using anti-GLI2 antibody (A), anti-GLI3 antibody (B) for immunoprecipitation, and anti-HA antibody for immunoblot. (C) Ubiquitination status of GLI2 and GLI3 following treatment of MCF-7 and MDA-MB-231 cells with IC_50_ dose of rolipram, H89, and co-treatment with H89 and rolipram for 18 hours. (D) Expressions of GLI2FL, GLI2R, and GLI3R in MCF-7 and MDA-MB-231 cells after treatment with TAK-243, co-treatment with TAK-243 and rolipram for 24 hours.

**Figure S5**


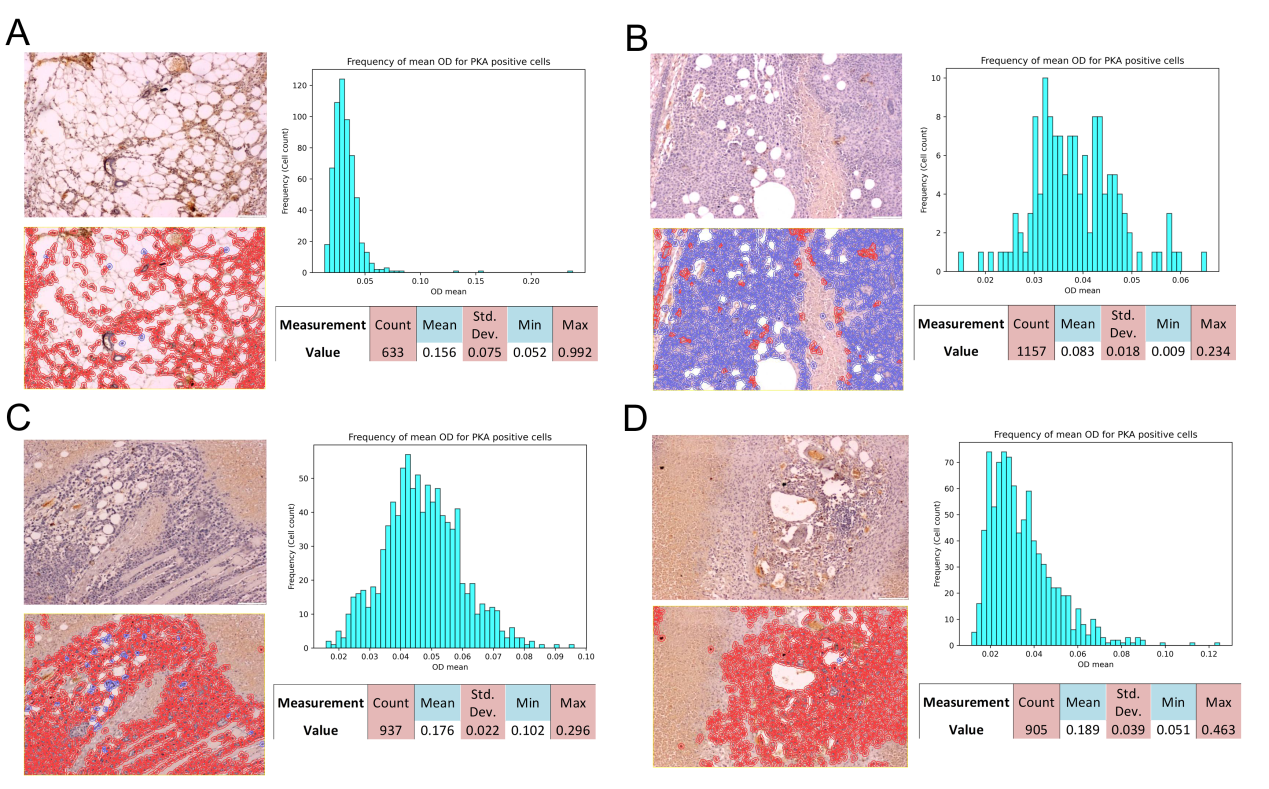


**Figure S5:** The upper left panels are the representative images of the expression of PKA in mammary fat pad of control mice (A), tumor-induced mice (B), mice treated with 1 mg/kg of rolipram (C), and mice treated with 5 mg/kg of rolipram (D), the lower left panels represent the output images from QuPath software, where red colored cells refer to the PKA-positive cells and the blue colored cells refer to PKA-negative cells. The upper right panels are the graphical representations of the mean optical density of the positive cells, and the lower right panels are the tabular representations of the values obtained from QuPath.

**Figure S6:**


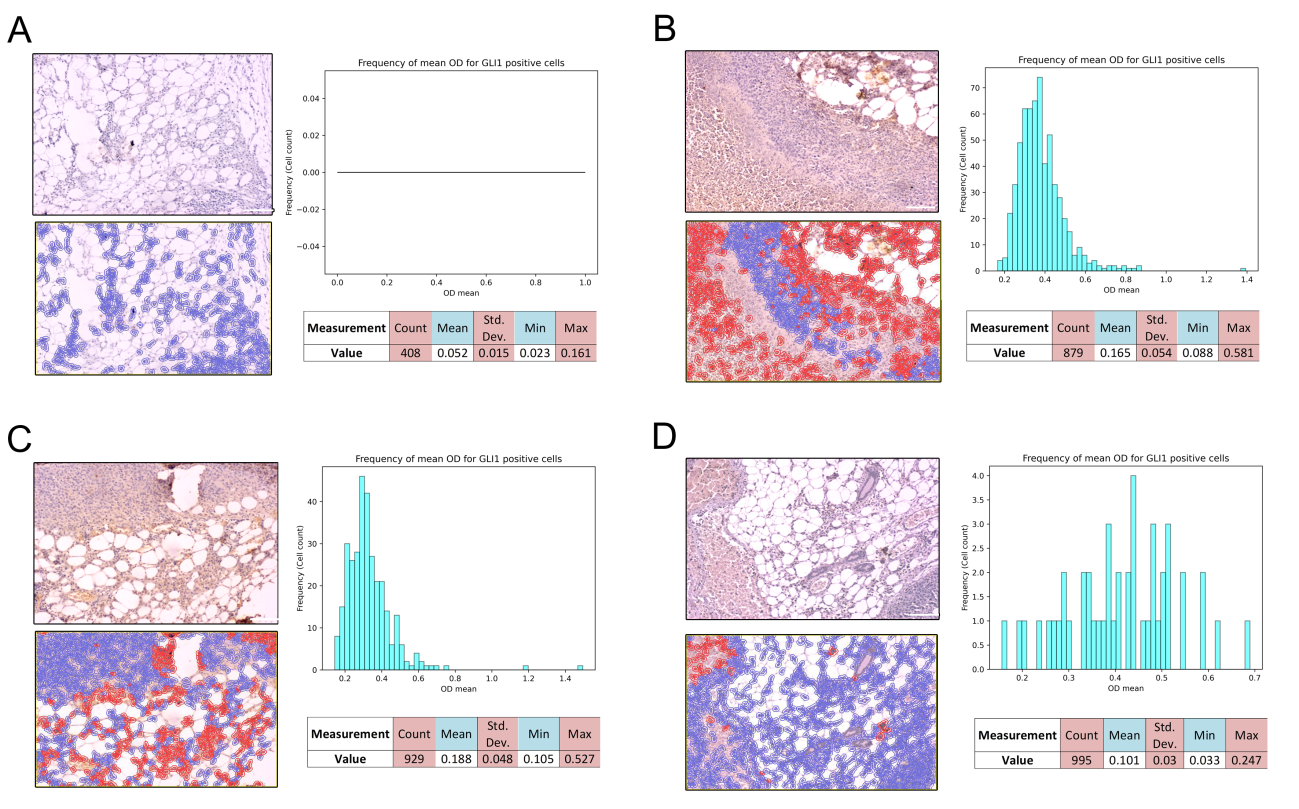


**Figure S6:** The upper left panels are the representative images of the expression of GLI1 in mammary fat pad of control mice (A), tumor-induced mice (B), mice treated with 1 mg/kg of rolipram (C), and mice treated with 5 mg/kg of rolipram (D), the lower left panels represent the output images from QuPath software, where red colored cells refer to the GLI1-positive cells and the blue colored cells refer to GLI1-negative cells. The upper right panels are the graphical representations of the mean optical density of the positive cells, and the lower right panels are the tabular representations of the values obtained from QuPath.

**Figure S7**


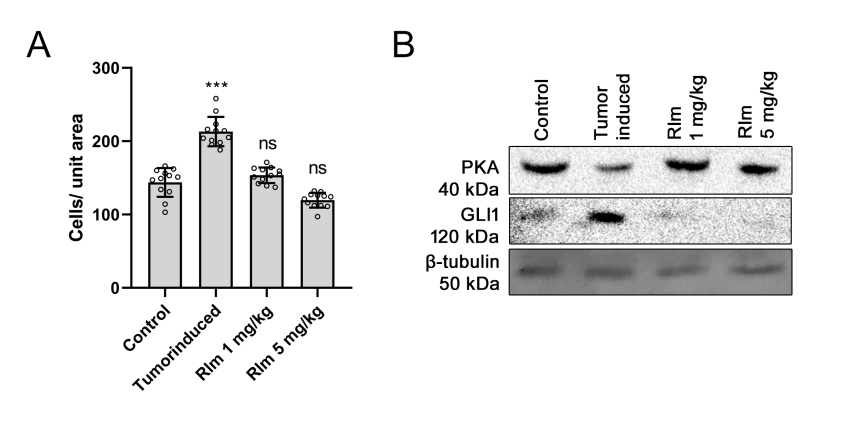


**Figure S7:** (A) Graphical representation corresponding to the number of cells present in a unit area (480 px × 400 px) in the histological sections of lung tissue from different groups of mice. (B) Western blots showing expressions of PKA and GLI1 in control, tumor-bearing mice, and tumor-bearing mice treated with 1 mg/kg body weight and 5 mg/kg body weight of rolipram.
